# Autocidal gravid ovitraps protect humans from chikungunya virus infection by reducing *Aedes aegypti* mosquito populations

**DOI:** 10.1371/journal.pntd.0007538

**Published:** 2019-07-25

**Authors:** Tyler M. Sharp, Olga Lorenzi, Brenda Torres-Velásquez, Veronica Acevedo, Janice Pérez-Padilla, Aidsa Rivera, Jorge Muñoz-Jordán, Harold S. Margolis, Stephen H. Waterman, Brad J. Biggerstaff, Gabriela Paz-Bailey, Roberto Barrera

**Affiliations:** 1 Centers for Disease Control and Prevention, Dengue Branch, San Juan, Puerto Rico, United States of America; 2 United States Public Health Service, Silver Springs, Maryland, United States of America; 3 Centers for Disease Control and Prevention, Division of Vector-Borne Diseases, Fort Collins, Colorado, United States of America; Fundaçao Oswaldo Cruz, BRAZIL

## Abstract

**Background:**

Public health responses to outbreaks of dengue, chikungunya, and Zika virus have been stymied by the inability to control the primary vector, *Aedes aegypti* mosquitos. Consequently, the need for novel approaches to *Aedes* vector control is urgent. Placement of three autocidal gravid ovitraps (AGO traps) in ~85% of homes in a community was previously shown to sustainably reduce the density of female *Ae*. *aegypti* by >80%. Following the introduction of chikungunya virus (CHIKV) to Puerto Rico, we conducted a seroprevalence survey to estimate the prevalence of CHIKV infection in communities with and without AGO traps and evaluate their effect on reducing CHIKV transmission.

**Methods and findings:**

Multivariate models that calculated adjusted prevalence ratios (aPR) showed that among 175 and 152 residents of communities with and without AGO traps, respectively, an estimated 26.1% and 43.8% had been infected with CHIKV (aPR = 0.50, 95% CI: 0.37–0.91). After stratification by time spent in their community, protection from CHIKV infection was strongest among residents who reported spending many or all weekly daytime hours in their community:10.3% seropositive in communities with AGO traps vs. 48.7% in communities without (PR = 0.21, 95% CI: 0.11–0.41). The age-adjusted rate of fever with arthralgia attributable to CHIKV infection was 58% (95% CI: 46–66%). The monthly number of CHIKV-infected mosquitos and symptomatic residents were diminished in communities with AGO traps compared to those without.

**Conclusions:**

These findings indicate that AGO traps are an effective tool that protects humans from infection with a virus transmitted by *Ae*. *aegypti* mosquitos. Future studies should evaluate their protective effectiveness in large, urban communities.

## Author summary

*Aedes* species mosquitos transmit pathogens of public health importance, including dengue, Zika, and chikungunya viruses. No tools exist to control these mosquitos that sustainably and effectively prevent human infections. Autocidal gravid ovitraps (AGO traps) have been shown to sustainably reduce *Aedes* populations by >80%. After chikungunya virus was introduced into Puerto Rico, we conducted serosurveys in communities with and without AGO traps. We observed a two-fold lower prevalence of chikungunya virus infection among residents of communities with AGO traps compared to communities without. Among infected residents of communities with traps, a significant proportion likely had been infected while outside their community. These findings indicate that AGO traps are an effective tool that protects humans from infection with pathogens transmitted by *Aedes* mosquitos.

## Introduction

Lack of sustainable and effective tools to control *Aedes aegypti* mosquito populations has resulted in the continued expansion of dengue virus (DENV) transmission worldwide and the recent emergence of chikungunya (CHIKV) and Zika (ZIKV) viruses in the western hemisphere [1, 2]. Despite the recent development of novel approaches to control *Ae*. *aegypti* mosquitos, most evaluations have relied on reductions in mosquito densities as the outcome measure as opposed to reductions in infections or disease in humans [1, 3, 4]. Moreover, those interventions that have evaluated both have detected decreases in mosquito abundance but not in human infections [5, 6]. The lack of effective approaches to reduce the disease burden attributable to these viruses is therefore driving the urgent search for novel vector control interventions [7–9]. However, evaluation of these novel approaches is complicated by the cyclical nature of epidemics and the inability to predict when they will occur, requiring that trials be conducted over multiple years in large populations [9]. Further complicating appropriate evaluation of vector control interventions is the need to measure human movement in and out of trial sites, which has been shown to be an important factor in DENV transmission within and between communities [10–12] that should be included to accurately measure the effectiveness of vector control interventions [3, 9, 12, 13].

The U.S. Centers for Disease Control and Prevention Dengue Branch (CDC-DB) recently developed an Autocidal Gravid Ovitrap (AGO trap) that suppresses adult *Ae*. *aegypti* mosquito populations by attracting and capturing gravid female mosquitos looking for a site to lay eggs [14]. During prospective community-based trials in Puerto Rico in 2011, communities with three AGO traps present outside of ~85% of homes resulted in a >80% reduction in adult *Ae*. *aegypti* mosquito populations compared to communities without AGO traps, differences that were sustained over more than three years with traps maintained by field staff [15–17].

After the first locally-acquired chikungunya cases in the Western Hemisphere were reported from the Caribbean in October 2013 [18], CHIKV rapidly spread throughout the Americas [19] and by the end of 2014 >1.7 million cases had been reported [20]. The first locally-acquired chikungunya case in Puerto Rico was detected in May 2014, and over the following year >28,000 suspected cases were reported [21]. In the communities where AGO traps have been under evaluation, the prevalence of CHIKV-infected mosquitos was significantly reduced in communities with AGO traps compared to those without traps [22]; however, the effect of the traps on limiting CHIKV transmission to community residents had not been epidemiologically evaluated.

Because chikungunya outbreaks often result in high rates of infection in humans (i.e., 38–63% [23]), and prior to its introduction the Puerto Rico population had no pre-existing immunity to CHIKV or related viruses [24, 25], the introduction of this virus provided the ideal situation to evaluate the effectiveness of AGO traps in preventing human infection with a virus transmitted by *Ae*. *aegypti* mosquitos. We report the results of a seroprevalence survey among residents of communities with and without AGO traps to determine their impact on CHIKV infection. Through a retrospective survey we also identified additional factors that may have affected residents’ risk of CHIKV infection, including demographic characteristics, human movement, and approaches to mosquito avoidance, and resulting illness and disability.

## Methods

### Ethics statement

This study was approved by CDC Institutional Review Board (protocol #6800). All adult participants provided written informed consent. Participants aged 15–20 years provided written asset and their parents or guardians provided written permission for study participation. Participants aged 5–14 years provided verbal assent and their parents or guardians provided written permission for study participation.

### Study site

Puerto Rico, an unincorporated territory of the United States, is the eastern-most island of the Greater Antilles archipelago, located in the northeastern Caribbean Sea. With an estimated population of 3.5 million in 2014 and a land mass of 3,515 square miles [26], Puerto Rico is the third most densely populated state or territory in the United States.

Evaluation of AGO traps has been ongoing since 2012 among four communities located in the municipalities of Salinas and Guayama, located on the southeastern coast (S1 Fig). The estimated populations of Salinas and Guayama in 2015 were 30,114 and 43,700, respectively, and land areas were 69.4 and 65.0 square miles (434 and 672 people per square mile). Median age in 2015 was 36.5 and 36.4 years, respectively, 15.5% and 14.6% of residents were aged ≥65 years, 48.6% and 49.6% of residents were male, and 56% and 53% of residents lived below the poverty line. Characteristics and satellite imagery of the four geographically separated communities in which AGO traps were evaluated are provided in S1 Fig and S1 Table.

### AGO trap use and mosquito surveillance

The design and methodology for use of AGO traps have been previously described [14, 16, 17, 27]. In summary, AGO traps consist of three primary components: a 19 liter black pail that contains hay and 10 liters of water to attract ovipositing female *Ae*. *aegypti* mosquitos; a capture chamber attached to the pail with a mesh cover that allows mosquitos to enter, and on the bottom a fine mesh that prevents mosquitos from reaching the water; and a sticky lining inside the chamber to which mosquitos adhere (S2 Fig). No insecticides are used in AGO traps. In the two intervention communities, three AGO traps are placed per home, typically in shaded areas outside but adjacent to homes (e.g., by doors, on the patio). AGO traps received bimonthly maintenance to replace water, hay, and the sticky lining.

Methods and results of mosquito surveillance in intervention and non-intervention communities during 2014 have been previously described [22]. In brief, mosquito surveillance traps were distributed approximately every 50 square meters throughout both communities with AGO traps (i.e., La Margarita and Villodas; “intervention communities”) and without AGO traps (i.e., Arboleda and La Playa; “non-intervention communities”) (S2 Table). Mosquitos were collected from surveillance traps weekly to monitor local populations of *Ae*. *aegypti*.

### Study design

Seroprevalence surveys were conducted during November 16, 2015 and January 16, 2016 to determine a possible difference in the prevalence of CHIKV infection among residents of intervention communities with AGO traps and non-intervention communities without traps. The population sizes for both intervention communities combined was 1,284, whereas that of both non-intervention communities combined was 1,218. Working under the assumptions that the populations had not significantly changed between 2010 and 2015, we calculated the number of individuals to sample before accounting for the cluster sampling design with anticipation of using the difference of two proportions estimated from finite populations as the comparative measure [28]. We assumed that the incidence of CHIKV infection in non-intervention communities was 15%, and computed the sample size needed to conclude that the incidence in intervention communities was half that (i.e., 7.5%), assuming power 80%, type I error α = 5%, and with equal allocation to the two populations. The resulting sample size was 178 individuals per group, or 356 participants. To account for the anticipated correlation induced by the cluster sampling, we assumed a design effect of two, resulting in an overall target sample size of 712 individuals.

We assigned a number to all structures in the community and randomly selected half of all structures in both intervention and non-intervention communities to attempt to offer household members study participation. Households were visited up to three times to attempt to offer all residents participation in the study. If the house was vacant or if the head-of-household was not available after the third visit, households were replaced until the target number of households had been offered enrollment.

Homes were neither included nor excluded from participation based on the presence of AGO traps. The head-of-household provided household-level information on characteristics of the household. Each participant completed an individual questionnaire that collected information on demographics, time spent in their community, mosquito avoidance behaviors, and recent illnesses, which was administered by study personnel. Parents or guardians responded to questionnaires as proxy for children <8 years of age.

All residents of the four communities participating in the ongoing evaluation of the AGO traps were eligible for inclusion in the seroprevalence survey. Individuals present but not residing in the communities (i.e., had slept in the household for fewer than four of the past seven nights) were excluded, as were children <5 years of age due to difficulty obtaining a blood specimen.

### Specimen testing

All blood specimens were transported to CDC-DB in San Juan, Puerto Rico, on the day of collection, centrifuged, and serum was aliquoted and frozen at -80°C. Serum specimens were tested by anti-CHIKV IgM and IgG ELISA [29, 30]. Participants whose serum specimen tested positive by either assay were defined as “CHIKV-positive”; all other participants were defined as “CHIKV-negative”.

As previously described [22], mosquitos were collected from surveillance traps weekly, identified to species and sex, pooled into groups of ≤20 female *Ae*. *aegypti*, transported to CDC-DB, and frozen at -80°C. Pools were later homogenized, and RNA was extracted for testing by RT-PCR to detect CHIKV and DENV RNA [31]. During the second half of 2014, a total of 1,334 pools were tested that included 26,251 individual female mosquitos. As previously reported, no mosquito pools were positive for DENV [22, 32–34].

### Data analysis

Similarity of survey participants to community residents was determined based on comparison with census data [26]. Analyses were adjusted by the cluster sampling design [35]. To ameliorate potential biases due to nonresponse, post-stratification by age group and sex was employed using census distributions as the reference [35, 36].

Demographic characteristics, time spent in their community, and mosquito avoidance behaviors of seroprevalence survey participants were compared between intervention and non-intervention communities as well as by status of CHIKV infection. Differences in observed proportions and medians were tested by applying the chi-squared test and the Mann-Whitney-Wilcoxon test, respectively.

Binomial generalized linear models (GLM) with the log link for survey data were used to calculate prevalence ratios (PR) and adjusted prevalence ratios (aPR) with corresponding 95% confidence intervals (CI) to determine the association of CHIKV infection with study variables and potential differences in these associations between intervention and non-intervention groups. Models included interaction terms with the intervention variable for demographics and behavioral characteristics. The models to estimate overall seroprevalence in intervention and non-intervention communities included interaction terms for age group, sex, and time spent at home. Due to computational issues resulting from insufficient data, and because no relevant changes in associations were observed when either age or sex were included, only time spent at home during daylight hours was included in models for other variables. Estimated medians for time spent at home for CHIKV-positive cases among residents of intervention and non-intervention communities were compared using Mood’s test.

GLM were also used to evaluate the association of illness and disability with status of CHIKV infection among participants from intervention and non-intervention communities. Adjustment variables included age, sex, and time spent at home during daylight hours. These variables were included for adjustment simultaneously, running one model for each risk factor characteristic using all the variables for adjustment at the same time. Disability was quantitated by querying participants for the days of work, school, or daily chores missed while they had fever with arthralgia and immediately afterwards, as well as the duration of fever, arthralgia, and arthritis. Estimated medians for duration of fever, arthralgia, and arthritis, duration of hospitalization, and number of days of work or daily chores missed were compared using Mood’s test.

To calculate the age-adjusted proportion of fever with arthralgia attributable to CHIKV infection, the frequency of reported fever with arthralgia among CHIKV-positive cases by age group (5–19, 20–59, or ≥ 60 years) was subtracted from the frequency of the same reported symptoms among CHIKV-negative cases [37]. The resulting frequencies were weighted by the number of individuals from each age group estimated to reside in the community and the number of study participants from each community. To depict the number of symptomatic CHIKV infections by month of reported illness onset since May 2014, in the event that participants reported multiple episodes of fever with arthralgia, the earliest reported date of illness onset was used.

Data were analyzed using the “survey” package from R software (V3.3.0, R Foundation for Statistical Computing, Vienna, Austria) and R-Studio Integrated Development Environment for R (R-Studio, Inc).

### Study data

De-identified data from the study reported herein are available in Supporting Information.

## Results

### Participant enrollment

**Fig 1:**
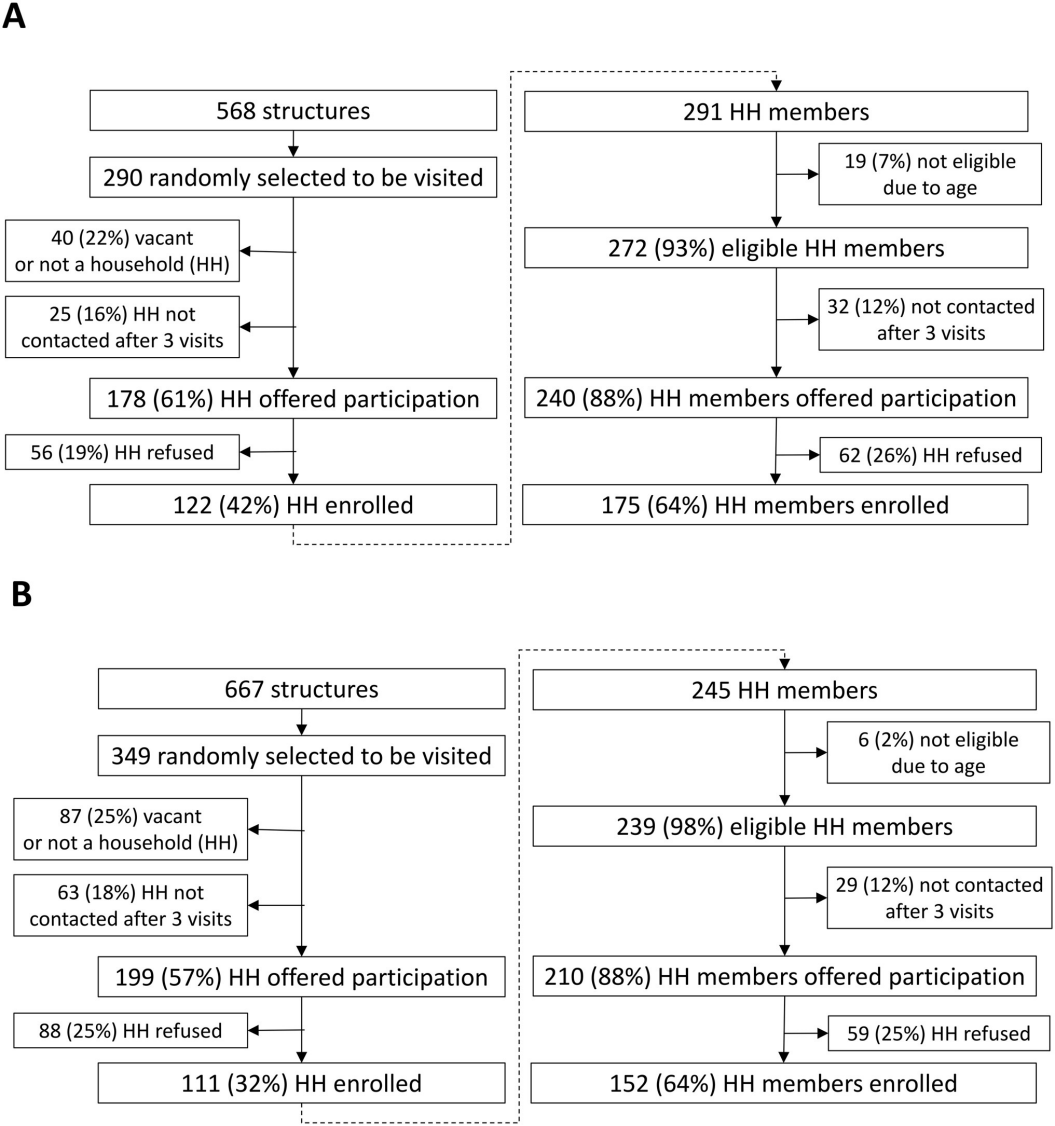
Enrollment of households and household residents in a survey of chikungunya virus seroprevalence among communities with (A) or without (B) autocidal gravid ovitraps (AGO traps) in Puerto Rico, November 2015–February 2016.

Of the 568 structures in intervention communities and 667 in non-intervention communities identified by satellite imagery, 290 (51.0%) and 349 (52.3%) structures, respectively, that were presumed to be households were randomly selected to be visited and offered participation in the seroprevalence survey (Fig 1). The proportions of selected structures that were not homes, were vacant homes, or were homes without a head-of-household able to be contacted were similar between intervention and non-intervention communities. Heads-of-household from 178 and 199 randomly-selected intervention and non-intervention community homes, respectively, were offered participation, and 122 (68.5%) and 111 (57.8%) accepted. Among 272 and 239 eligible residents from intervention and non-intervention community households, respectively, 175 (64.3%) and 152 (63.6%) were enrolled in the seroprevalence survey.

Most household characteristics did not differ significantly between intervention and non-intervention communities or between study participants and community residents; however, study participants from both intervention and non-intervention communities were slightly but significantly older than all residents (S3 and S4 Tables). When comparing participants in intervention versus non-intervention communities, non-intervention community participants were slightly but significantly older and more often female than intervention community participants (Table 1). Non-intervention community participants more frequently reported being retired (28.7% vs. 21.1%, *P* = 0.0123), whereas intervention community participants more often reported being employed or in school (63.9% vs. 55.3%, *P* = 0.0290). Non-intervention community participants reported being home during daylight hours more often than intervention community members (76 vs. 63 hours per week, *P* = 0.0001), and more frequently reported being bitten by mosquitos either daily (53.6% vs. 30.3%, *P* = 0.0009) or weekly (19.6% vs. 11.0%, *P* = 0.0268); intervention community participants more frequently reported rarely being bitten by mosquitos (54.5% vs. 23.9%, *P <* 0.0001). Non-intervention community participants more frequently reported being bitten by mosquitos during the morning (16.9% vs. 5.4%, *P* = 0.0005) and daytime (31.0% vs. 12.1%, *P* = 0.0007), and more frequently reported being bitten by mosquitos while at home (80.5% vs. 70.7%, *P* = 0.0244) and school or work (11.4% vs. 4.1%, *P* = 0.0073). Non-intervention community participants also reported more frequent daily use of mosquito repellent (23.7% vs. 6.3%, *P* < 0.0001) and sleeping under a bed net (8.9% vs. 1.2%, *P* = 0.0002).

**Table 1:** Comparison of demographic characteristics and reported behaviors among participants of a survey of chikungunya virus seroprevalence among communities with (intervention) or without (non-intervention) autocidal gravid ovitraps (AGO traps) in Puerto Rico, November 2015–February 2016.

Characteristics	Intervention community participants N = 175 n (column %)	Non-intervention community participants N = 152 n (column %)	*P* value*
**Demographics**			
Age, median (range)	55 (5–89)	58 (6–86)	**0.0001**^†^
Age group			
5–19	13 (7.4)	10 (6.6)	**<0.0001**
20–49	50 (28.6)	34 (22.4)	**<0.0001**
50+	112 (64.0)	108 (71.1)	**<0.0001**
Female sex^‡^	112 (50.5)	93 (52.9)	**<0.0001**
Years living in community, median (range)	20 (0–70)	24 (0–76)	0.1076^†^
Employment status^‡^			
Unemployed	45 (15.0)	36 (16.0)	0.7939
Retired	73 (21.1)	68 (28.7)	**0.0123**
Working/In school	56 (63.9)	48 (55.3)	**0.0290**
**Behaviors and mosquito activity**			
Weekly hours at home during daylight hours, median (range)	63 (14–84)	76 (0–84)	**0.0001**^†^
Frequency of mosquito bites^‡^			
Never	12 (4.2)	6 (2.9)	0.4705
Rarely	92 (54.5)	52 (23.9)	**<0.0001**
Weekly	21 (11.0)	23 (19.6)	**0.0268**
Daily	46 (30.3)	66 (53.6)	**0.0009**
Time of day when mosquitos bite^‡^			
Morning	17 (5.4)	30 (16.9)	**0.0005**
Daytime	28 (12.1)	45 (31.0)	**0.0007**
Evening	88 (51.9)	86 (50.3)	0.7874
Night	68 (43.4)	51 (35.9)	0.2867
Never	13 (4.3)	4 (1.6)	0.0628
Where bitten by mosquitos^‡^			
Home	123 (70.7)	127 (80.5)	**0.0244**
School or work	5 (4.1)	7 (11.4)	**0.0073**
Others’ homes inside my community	11 (3.8)	13 (5.7)	0.2963
Others’ homes outside my community	17 (14.1)	21 (13.6)	0.9118
Other places	33 (20.8)	21 (15.1)	0.1205
Mosquitos never bite me	12 (4.1)	3 (1.1)	**0.0224**
Frequency of use of mosquito repellent^‡^			
Never	66 (39.8)	48 (29.1)	0.0882
Daily	15 (6.3)	31 (23.7)	**<0.0001**
Occasionally	92 (53.9)	73 (47.2)	0.3543
Frequency of sleeping under bed net^‡^			
Never	171 (98.8)	139 (91.1)	**0.0002**
Daily	4 (1.2)	13 (8.9)	**0.0002**

Abbreviations: CHIKV = chikungunya virus; RR = relative risk; 95% CI = 95% confidence interval

*Chi-square test

^†^Wilcoxon Test for comparison between medians

^‡^Proportions shown are estimates for the community based on survey responses

### Association of CHIKV seroprevalence with AGO traps

Among the 327 participants, a total of 114 (34.9%) had serologic evidence of CHIKV infection: 81 (71.1%) were positive by IgG ELISA only, 28 (24.6%) by both IgM and IgG ELISA, and 5 (4.4%) by IgM ELISA only. The unadjusted rate of CHIKV seropositivity among intervention and non-intervention community participants was 25.1% and 46.1%, respectively (Table 2). After weighting for differences in age and sex between study participants and population census data, the estimated seroprevalence among residents of non-intervention and intervention communities was 26.1% and 43.8%, respectively (PR = 0.60, 95% CI 0.44–0.81).

**Table 2:** Proportion of residents with serologic evidence of chikungunya virus infection from communities with (intervention) or without (non-intervention) autocidal gravid ovitraps (AGO traps) by demographic and behavioral characteristics, Puerto Rico, November 2015–February 2016.

	Intervention Communities			Non-intervention Communities			PR (95% CI)	aPR^†^ (95% CI)
	N	CHIKV+ Survey Participants, n (row %)	Estimated Proportion CHIKV+ in Community*	N	CHIKV+ Survey Participants, n (row %)	Estimated Proportion CHIKV+ in Community*		
Overall	175	44 (25.1)	26.1	152	70 (46.1)	43.8	**0.60 (0.44–0.81)**	**0.50 (0.37–0.91)**^‡^
Age group								
5–19	13	5 (38.5)	32.8	10	4 (40.0)	47.9	**0.68 (0.47–0.99)**	**0.68 (0.48–0.97)**
20–49	50	15 (30.0)	27.0	34	13 (38.2)	39.7	0.68 (0.36–1.29)	**0.54 (0.33–0.89)**
50+	112	24 (21.4)	17.2	108	53 (49.1)	44.7	0.39 (0.25–0.59)	**0.34 (0.22–0.55)**
Sex								
Male	63	17 (27.0)	25.9	59	25 (42.4)	29.5	0.88 (0.53–1.46)	0.90 (0.55–1.48)
Female	112	27 (24.1)	26.3	93	45 (48.4)	56.5	**0.47 (0.32–0.68)**	0.86 (0.52–1.43)
Weekly hours at home during daylight hours, median (range or 95% CI)	175	63 (14–84)	43.5 (39.0–55.8)	151	76 (0–84)	49.0 (44.5–56.0)	**0.0015**^§^	
1–24	14	5 (35.7)	40.6	10	4 (40.0)	40.4	1.01 (0.39–2.58)	--
25–60	69	26 (37.7)	31.3	52	21 (40.4)	40.7	0.77 (0.53–1.11)	--
61–84	92	13 (14.1)	10.3	89	45 (50.6)	48.7	**0.21 (0.11–0.41)**	--
Frequency of use of mosquito repellent since May 2014								
Never	66	16 (24.2)	25.3	48	21 (43.8)	40.2	0.63 (0.36–1.10)	0.63 (0.36–1.09)
Daily	15	5 (33.3)	33.3	31	14 (45.2)	53.9	0.62 (0.28–1.37)	0.84 (0.36–1.95)
Occasionally	92	22 (23.9)	25.3	73	35 (47.9)	40.9	0.62 (0.34–1.11)	0.62 (0.35–1.10)

Abbreviations: CHIKV = chikungunya virus; PR = prevalence ratio; aPR = adjusted prevalence ratio; 95% CI = 95% confidence interval; -- = PR could not be adjusted for the indicated variable

*Estimates for the entire community based on survey responses by age group and sex.

^†^Adjusted only by time spent at home during daylight hours

^‡^Adjusted by age, sex, and time spent at home during daylight hours

^§^Mood’s test for medians

**Fig 2:**
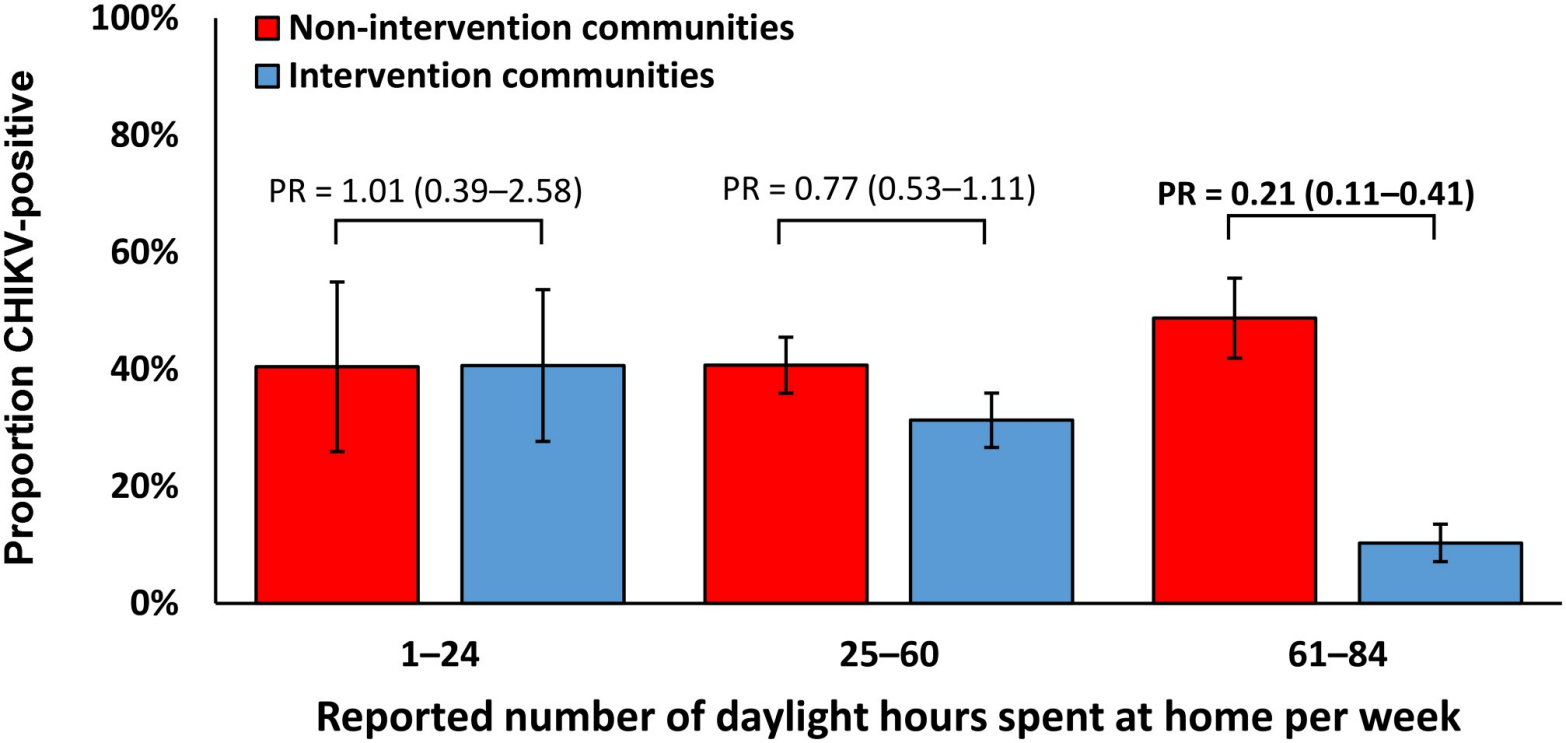
Estimated proportion of chikungunya virus infected residents of communities in Puerto Rico with (intervention; n = 175) or without (non-intervention; n = 152) autocidal gravid ovitraps (AGO traps) by reported number of daylight hours spent at home per week, November 2015–February 2016. Abbreviations: PR = prevalence ratio; error bars indicate standard error.

To determine if factors other than residence in intervention or non-intervention communities may have been responsible for the observed association of intervention communities with decreased prevalence of CHIKV infection, we compared CHIKV seroprevalence in intervention and non-intervention communities by selected demographic and behavioral characteristics (Table 2). We first observed that magnitude of protection from CHIKV infection in intervention vs. non-intervention communities differed by time spent in residents’ communities (Fig 2). Although estimated seroprevalence of CHIKV infection was not significantly different between intervention and non-intervention community residents who spent 1–24 or 25–60 daylight hours per week at home or in their community, we observed a difference in estimated seroprevalence between residents of intervention communities and residents of non-intervention communities who spent 61–84 daylight hours per week at home or in their community (10.3% vs. 48.7%, respectively; PR = 0.21, 95% confidence interval [CI]: 0.11–0.41). Unadjusted estimates are presented as the model did not converge when adjusting by sex or age.

After adjusting for time spent in residents’ communities during daylight hours, the adjusted prevalence ratio (aPR) of CHIKV infection among residents of intervention as compared to non-intervention communities was 0.50 (95% CI: 0.37–0.91) (Table 2). No significant differences were observed among residents of intervention and non-intervention communities in adjusted prevalence of CHIKV infection by sex or reported frequency of use of mosquito repellent. The magnitude of protection from CHIKV infection among residents of intervention compared to non-intervention communities increased with age.

### Factors associated with CHIKV infection

We next evaluated for association of demographic, environmental, and behavioral characteristics with CHIKV seropositivity among residents of intervention and non-interventions communities combined, including evaluation for interaction between participants’ status of residence in an intervention versus non-intervention community with the evaluated variables. Neither age, employment status, frequency of use of mosquito repellent or sleeping under a bed net, nor household characteristics including annual income, presence of window screens, or use of air conditioning were significantly associated with CHIKV infection (Table 3). A greater estimated proportion of females were seropositive compared to males (*P* = 0.0018); however, interaction with status of intervention was observed (*P* = 0.0335), and increased risk of seropositivity was only significant among residents of non-intervention communities (intervention communities: 26.3% seropositivity in females vs. 25.9% in males, RR = 1.02 [95% CI: 0.66–1.55]; non-intervention communities: 56.5% seropositivity in females vs. 29.5% in males, RR = 1.91 [95% CI: 1.28–2.86]). Living in a two-story home was associated with decreased seroprevalence (*P* = 0.0072); however, there was significant interaction with status of intervention (*P* = 0.0057), and significant association was only observed among residents of non-intervention communities (intervention communities: 38.7% seropositivity in residents of two-story homes vs 25.3% in residents of one-story homes, RR = 1.53 [95% CI: 0.64–3.66]; non-intervention communities: 7.2% seropositivity in residents of two-story homes vs 45.0% in residents of one-story homes, RR = 0.16 [95% CI: 0.04–0.60]). Reported frequency of mosquito bites was significantly associated with CHIKV infection such that increased frequency of reported bites was associated with increased prevalence of CHIKV infection (*P* = 0.0056); however, here as well significant interaction with status of intervention was observed (*P* = 0.0072), and the association was only observed among residents of non-intervention communities. Reporting mosquito bites outside of their community (*P* = 0.0013) or elsewhere (*P* = 0.0002) were both significantly associated with CHIKV infection in the absence of detectable interaction with intervention status. Similarly, reporting being bitten by mosquitos at school or work was significantly associated with protection from CHIKV infection (*P* < 0.0001) in the absence of detectable interaction.

**Table 3:** Demographic and behavioral characteristics associated with chikungunya virus infection among survey participants from four communities in Puerto Rico, November 2015–February 2016.

Characteristics	All survey participants N = 327 n (column %)	CHIKV+ survey participants N = 114 n (row %)	Estimated Proportion CHIKV+ in Community*	*P* value^†^
**Demographics**				
Age group				0.6161
5–19	23 (7.0)	9 (39.1)	40.8	
20–49	84 (25.7)	28 (33.3)	34.2	
≥50	220 (67.3)	77 (35.0)	35.2	
Sex				**0.0018**^‡^
Male	205 (62.7)	42 (34.4)	28.0	
Female	122 (37.3)	72 (35.1)	44.3	
Status of employment				0.7676^†^
Unemployed	81 (24.8)	29 (35.8)	33.4	
Retired	141 (43.1)	47 (33.3)	36.6	
Working/studying	104 (31.8)	38 (36.5)	37.3	
**Housing characteristics**				
Housing type				**0.0072**^‡^
1-story	300 (91.7)	107 (35.7)	36.6	
2-story	24 (7.3)	5 (20.8)	20.2	
Other	3 (0.9)	2 (66.7)	64.5	
Intact screens on windows and doors				0.4357
None	64 (19.6)	34 (53.1)	41.8	
Some	116 (35.5)	43 (37.1)	38.3	
All	147 (45.0)	37 (25.2)	32.1	
Air conditioning use				0.2175
Never	114 (34.9)	46 (40.4)	37.6	
Ever	213 (65.1)	68 (31.9)	36.1	
Leave doors/windows open				0.2194^‡^
Never	81 (24.8)	28 (34.6)	35.9	
Ever	246 (75.2)	86 (35.0)	36.6	
Use citronella candles or mosquito coil	90 (27.5)	35 (38.9)	35.9	0.4690
Annual income				0.1544
<$25,000	202 (61.8)	73 (36.1)	34.6	
$25,000–$50,000	63 (19.3)	20 (31.7)	34.5	
>$50,000	12 (3.7)	5 (41.7)	53.1	
Decline to respond	50 (15.3)	16 (32.0)	40.4	
**Mosquito activity and avoidance behaviors**				
Frequency of mosquito bites				**<0.0001**^‡^
Never	18 (5.5)	7 (38.9)	52.0	
Rarely	144 (44.0)	46 (31.9)	26.6	
Weekly	44 (13.5)	16 (36.4)	38.3	
Daily	112 (34.2)	43 (38.4)	42.1	
Time when mosquitos bite				
Morning	47 (14.4)	20 (42.6)	41.0	0.5571^‡^
Daytime	73 (22.3)	30 (41.1)	38.7	0.6193
Evening	174 (53.2)	60 (34.5)	37.8	0.2778
Night	119 (36.4)	39 (32.8)	37.5	0.5543
Never	17 (5.2)	5 (29.4)	40.1	0.0013
Where bitten by mosquitos				
Home	250 (76.5)	91 (36.4)	36.4	0.5997
School or work	12 (3.7)	0 (0)	0	**<0.0001**
Others’ homes inside my community	24 (7.3)	8 (33.3)	26.7	0.2210
Others’ homes outside my community	38 (11.6)	22 (57.9)	55.6	**0.0140**
Elsewhere	54 (16.5)	23 (42.6)	57.1	**0.0066**
Never	15 (4.6)	3 (20.0)	30.1	0.0862
Frequency of use of mosquito repellent				0.3202
Never	114 (34.9)	37 (32.5)	32.9	
Daily	46 (14.1)	19 (41.3)	50.7	
Occasionally	165 (50.5)	57 (34.5)	33.9	
Frequency of sleeping under bed net				0.4965
Never	310 (94.8)	105 (33.9)	35.6	
Daily	17 (5.2)	9 (52.9)	49.9	

Abbreviations: CHIKV+ = Positive for chikungunya virus infection

*Estimates for the entire community based on survey responses by age group and sex. Comparison group is the estimated proportion CHIKV- in community.

^†^Adjusted by age, sex, time spent at home during daylight hours, and frequency of use of repellent

^‡^Significant interaction exists between status of intervention and the indicated variable

### Illness and disability associated with CHIKV infection

Frequency of reported history of arthralgia prior to May 2014 (when CHIKV was first detected to be circulating in Puerto Rico) did not differ among CHIKV-positive and CHIKV-negative participants; however, CHIKV-positive participants were five-fold more likely to report having experienced fever and arthralgia after May 2014 (adjusted relative risk [aRR] = 5.3, 95% CI: 3.6–7.9) (Table 4). Among 114 CHIKV-positive and 213 CHIKV-negative participants, 84 (73.7%) and 32 (15.0%), respectively, reported having experienced fever and arthralgia since May 2014. The estimated proportion of fever with arthralgia attributable to CHIKV infection (i.e., rate of symptomatic infection) among participants aged 5–19, 20–49, and ≥50 years was 51% (95% CI: 24–68%), 68% (95% CI: 45–81%), and 54% (95% CI: 39–66%), respectively. The age-adjusted proportion of fever with arthralgia attributable to CHIKV infection was 58% (95% CI: 46–66%).

**Table 4:** Association of illnesses and disability with chikungunya virus infection among residents of four communities in Puerto Rico, November 2015–February 2016.

Characteristics	CHIKV+ survey participants N = 114 n (column %)	CHIKV- survey participants N = 213 n (column %)	Estimated Proportion CHIKV+ in Community	Estimated Proportion CHIKV- in Community	RR (95% CI)	aRR (95% CI)
History of joint pain prior to May 2014*	29 (25.4)	61 (28.6)	19.0	18.8	1.0 (0.7–1.5)	0.9 (0.6–1.4)
Fever and arthralgia since May 2014*	84 (73.7)	32 (15.0)	77.8	18.8	**5.2 (3.4–7.9)**	**5.3 (3.6–7.9)**
Duration of fever in days, median (range or 95% CI)	3 (1–14)	3 (1–14)	4.0 (3.0–5.7)	4.0 (2.7–4.0)	0.9610^†^	
Duration of arthralgia in days, median (range or 95% CI)	14 (2–570)	5 (1–365)	14.0 (14–30)	3.0 (3.0–6.0)	**<0.0001**^†^	
Arthritis since May 2014*	27 (23.7)	23 (10.8)	25.7	6.2	**2.2 (1.8–2.9)**	**2.2 (1.7–2.8)**
Duration in days, median (range or 95% CI)	90 (3–420)	30 (30–99)	90.0 (24.5–118.7)	30.0 (27.9–55.9)	**0.0124**^†^	
Times sought medical care	57 (50.0)	20 (9.4)	70.6	76.7	0.9 (0.7–1.2)	1.0 (0.7–1.2)
Once	28 (24.6)	9 (4.2)	44.2	57.9	NA	
More than once	28 (24.6)	11 (5.2)	55.8	42.2	1.2 (0.9–1.6)	1.1 (0.8–1.4)
Hospitalized	4 (3.5)	5 (2.4)	4.0	20.5	0.4 (0.2–1.0)	**0.4 (0.2–0.8)**
Duration in days, median (range or 95% CI)	9 (2–10)	5 (1–12)	9.0 (1.3–10.0)	5.6 (0.2–10.9)	0.3209^†^	
Missed work or daily chores since May 2014*	51 (44.7)	39 (18.3)	48.0	16.1	**2.4 (1.7–3.3)**	**2.4 (1.7–3.2)**
Days of work/chores lost, median (range or 95% CI)	7 (1–90)	5.5 (0–120)	6.6 (3.0–7.0)	13.2 (4.5–19.3)	0.5331^†^	

Abbreviations: CHIKV = chikungunya virus; RR = relative risk; 95% CI = 95% confidence interval; aPR = adjusted relative risk (adjusted for age group, sex, and time spent at home); NA = not applicable

*Chikungunya virus was first detected to be circulating in Puerto Rico in May, 2014

^†^*P* value, Mood’s test for medians

**Fig 3:**
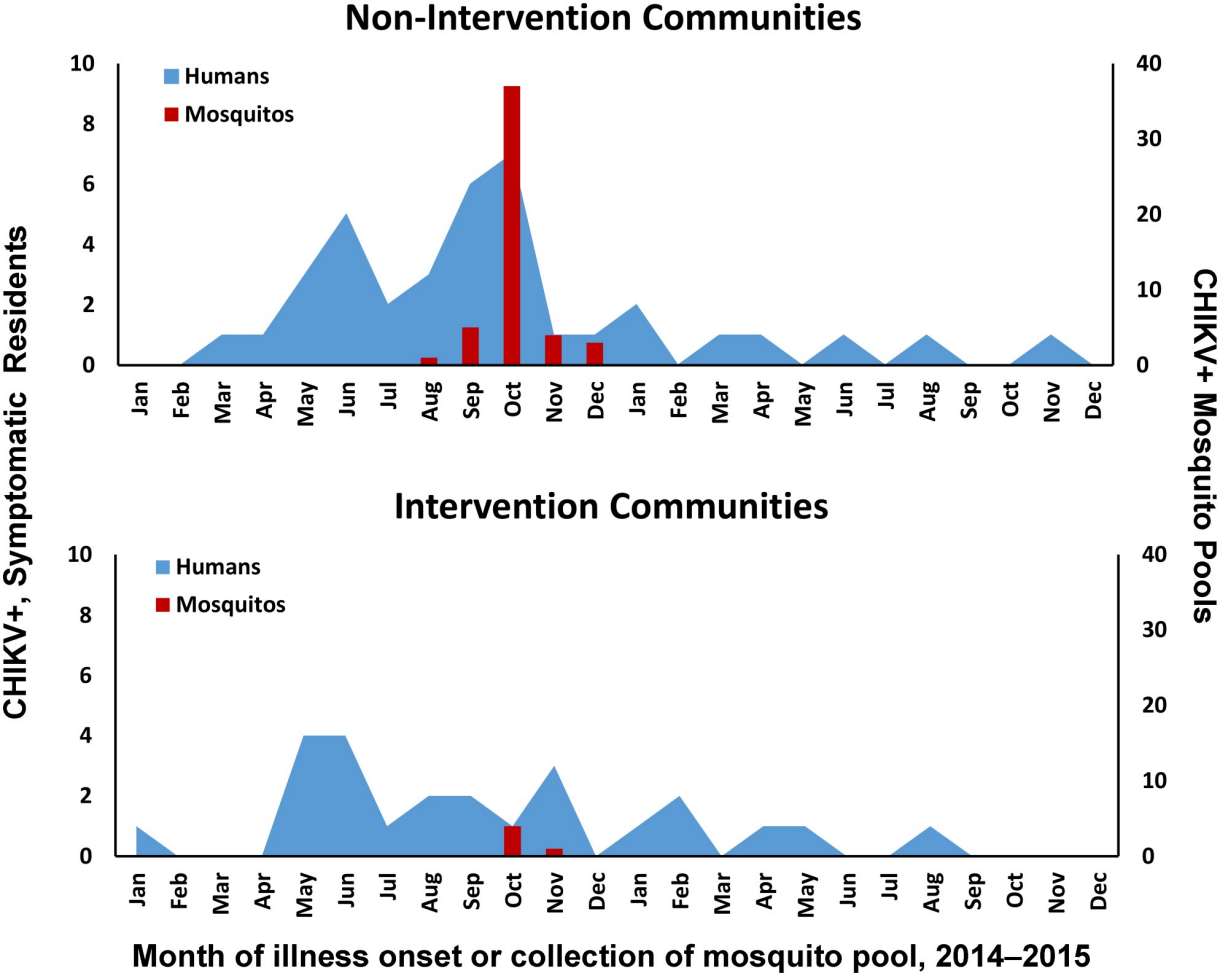
Number of participants with evidence of chikungunya virus infection who reported fever with arthralgia by month and year of illness onset (N = 81*), and number of mosquito pools in which chikungunya virus RNA was detected by RT-PCR (N = 50) from communities with (intervention) or without (non-intervention) autocidal gravid ovitraps (AGO traps) in Puerto Rico, November 2015–February 2016. *As reported during the serosurvey; 3 participants had unknown timing of illness onset.

The first participant with evidence of symptomatic CHIKV infection reported illness onset in January 2014 and resided in an intervention community (Fig 3). No additional symptomatic CHIKV infections were identified among study participants from intervention communities until May 2014, after which case counts increased to a maximum of four cases per month for two consecutive months and decreased but continued at comparatively low levels until the last identified case had illness onset in August 2015. In contrast, cases among participants from non-intervention communities steadily increased after the first case was detected in March 2014 until the peak (n = 7) was detected in October 2014, after which case counts decreased appreciably but continued at low levels through November 2015. The monthly number of human chikungunya cases in intervention and non-intervention communities followed similar trends as CHIKV RNA-positive mosquito pools. CHIKV RNA was detected in 5 of 169 (3.0%) and 50 of 1,165 (5.0%) mosquito pools collected from surveillance traps in intervention and non-intervention communities, respectively, such that the expected number of infected mosquitos per trap per week was roughly ten-fold smaller in intervention as compared to non-intervention communities [22, 38].

Although the reported duration of fever did not differ among CHIKV-positive compared to CHIKV-negative participants, the reported duration of arthralgia was significantly longer among CHIKV-positive participants (median = 14 vs. 5 days; *P* = 0.0005) (Table 4). Similarly, CHIKV-positive individuals were twice as likely to report arthritis (aRR = 2.2, 95% CI: 1.7–2.8), which lasted significantly longer among CHIKV-positive participants (median = 90 vs. 30 days; *P* = 0.0124). Although frequency of seeking medical care and the number of times individuals sought care were not significantly different between participants with and without CHIKV infection, CHIKV-positive participants were less likely to be hospitalized (aRR = 0.4, 95% CI: 0.2–0.8). CHIKV-positive participants also reported having missed work or daily chores due to their illness more than twice as often as CHIKV-negative participants (aRR = 2.4, 95% CI: 1.7–3.2).

## Discussion

The introduction of CHIKV into the Americas provided a unique opportunity to evaluate an ongoing study of AGO traps in Puerto Rico and determine their effectiveness in preventing CHIKV infection in humans. Serologic evidence of CHIKV infection was detected among roughly one-quarter of residents of communities with AGO traps and one-half of residents of communities without AGO traps. After accounting for differences in time spent in residents’ communities, the presence of AGO traps was still protective against CHIKV infection. This study extends observed reductions in rates of CHIKV infection in mosquitos [22, 32–34], and is the first to show reduced rates of human infection with a pathogen transmitted by *Ae*. *aegypti* with the use of a novel vector control tool.

Throughout various levels of analysis, residence in communities in which AGO traps were present remained significantly associated with protection from CHIKV infection. However, level of protection by AGO traps was most strongly affected by time spent in study participants’ community of residence. Participants that reported being present in communities with AGO traps during a majority of weekly daylight hours, when *Ae*. *aegypti* are more likely to bite [39], were most strongly protected from CHIKV infection (i.e., five-fold lower seroprevalence). This finding also supports recommendations that human mobility should be incorporated into evaluations of the effectiveness of vector control interventions in reducing human infections [3, 9, 12, 13]. Though retrospectively collected, the lower apparent number of chikungunya cases among communities with AGO traps further supports the association of AGO traps with prevention of CHIKV transmission.

We observed that CHIKV infection was significantly associated with reporting mosquito bites at locations outside of residents’ communities among residents of communities with and without AGO traps. This finding is consistent with importation of CHIKV into communities via infected humans due to human movement in and out of communities, a recognized and major contributor to DENV dissemination both within and between communities [10].

Although the timing of apparent CHIKV transmission fits well with observed patterns from throughout Puerto Rico [40], several infected individuals reported having had onset of fever and arthralgia before the first chikungunya case was identified in Puerto Rico in early May 2014 [21]. Potential explanations for this observation include: circulation of CHIKV in these communities before the first confirmed case was detected [41]; CHIKV-infected individuals having misreported the month or year in which their illness occurred; or, individuals having had fever with arthralgia due to another etiology prior to May 2014 and being infected with CHIKV thereafter.

The epidemiologic characteristics associated with CHIKV infection as well as the illness observed in this population were generally consistent with findings from previous studies [42–44]. The observed differences in the frequency, timing, and location of reported mosquito bites between community members with and without AGO traps are consistent with a diminished presence in the intervention communities of *Ae*. *aegypti* mosquitos. Similarly, residents of communities without AGO traps reported more frequent use of mosquito repellent and bed nets. These findings together suggest that due to reduced presence of *Ae*. *aegypti* in communities with AGO traps, residents less frequently employed alternative approaches to avoid mosquito bites. Although females from communities without AGO traps were more often infected with CHIKV, this finding should be interpreted with caution as seroprevalence and seroincidence surveys have variably observed both males and females to be at increased risk for infection with CHIKV, DENV, or ZIKV [43, 45]. We also did not observe an association of CHIKV infection with age, which was recently reported from a pediatric cohort study in Nicaragua [46]. These disparities may be the result of differences in study design and/or community or culture-specific differences in exposure to *Ae*. *aegypti* mosquitos.

The proportion of fever with arthralgia attributable to CHIKV observed in this study (58%) is similar to previously reported ratios of symptomatic-to-asymptomatic CHIKV infection in Puerto Rico [44] and elsewhere [43, 45, 47]. Although mortality associated with CHIKV infection is rare [48–51], multiple studies have reported that debilitating arthralgia and/or arthritis may occur for weeks or months after illness onset [23, 52–55]. We also observed this in our study population, as nearly three-quarters of infected individuals reported arthralgia that lasted a median of two weeks and nearly one-quarter reported arthritis that lasted for 90 days or more. These manifestations together resulted in nearly half of infected individuals reporting having lost a median of seven days of work or daily chores. In this population-based study, the frequency and duration of debilitating joint disease associated with CHIKV infection was lower than that reported in other studies [52, 53, 55]. A potential explanation for these discrepancies is that the data collected in our study were population-based, as opposed to previous studies that reported disability among a selection of ill individuals who sought medical care, which may have introduced selection bias.

Because no vaccines are currently available to prevent chikungunya [56] or ZIKV infection [57], and the only commercially produced dengue vaccine is only partially protective [58, 59], mosquito control remains important for primary prevention of infection with pathogens transmitted by *Ae*. *aegypti*. This objective is unlikely to be achieved on a population level through current practices using insecticides or larvicides [1, 2]. Novel approaches to mosquito control currently under evaluation include release of modified *Ae*. *aegypti* mosquitos that reduce mosquito populations or compromise their capacity for pathogen transmission, toxic sugar baits, devices to mediate autodissemination of larvicide, training and organization of community members to eliminate mosquito breeding sites with quantitative feedback using social media, and, as demonstrated herein, mosquito traps [60, 61]. As all such approaches have strengths and weaknesses, combining approaches may yield the greatest impact.

The strengths of this study include comparison of prevalence (which effectively was the incidence due to no underlying immunity) of CHIKV infection as defined by serologic diagnostic testing in four demographically, environmentally, and geographically similar communities with years-long surveillance of adult *Ae*. *aegypti* populations. Nonetheless, our study was subject to several limitations. First, the four communities were not randomly selected to have AGO traps placed in homes. Consequently, though unlikely, the observed differences in seroprevalence of CHIKV infection may be attributable to a factor(s) other than the presence of AGO traps (e.g., community-specific changes in prevalence of mosquito breeding sites during the study period, vector competence, or development of antibodies to CHIKV infection). Second, questionnaire data were self-reported and collected retrospectively, and hence subject to recall bias. This potential bias may have resulted in data inaccuracies and either over- or under-estimation of pertinent variables (e.g., date of illness onset, time spent at home), deficiencies that may have been ameliorated had data been collected prospectively (e.g., fever diaries, GPS units to track human movement [10, 62]). Due to this limitation as well as lack of collection of the number of night-time hours that participants spent outside of their home, limited sample size, and not being able to rule-out infection at work or school while in participants’ communities, we were unable to estimate the number of CHIKV infections among residents of communities with AGO traps present that occurred as a result of intracommunity transmission. In addition, clinical signs and symptoms were reported by study participants, some of which would have been more accurately evaluated by a clinician (e.g., arthritis). Last, although the effectiveness of use of AGO traps on a broad scale is unknown, results from a recent evaluation of AGO traps in combination with community education, source reduction, and application of larvicide in a large, urban setting demonstrated reductions in *Ae*. *aegypti* populations similar to those observed in smaller communities [63]. Should the requirement for bimonthly maintenance of AGO traps limit their implementation on a larger scale, recent evaluations have shown that AGO traps can also be used for rapid focal reductions in mosquito populations around target households [32]. Such a strategy may be optimal for short-term protection among high-risk individuals (e.g., protection of pregnant women from ZIKV infection) or long-term protection in areas with high risk for transmission and resources available for trap maintenance (e.g., schools) [9].

In summary, by conducting a survey to estimate the seroprevalence of CHIKV infection among residents of communities with and without the AGO traps that had been under study for several years prior to the introduction of CHIKV, we observed that the presence of the traps was strongly associated with protection from CHIKV infection. We expect that AGO traps would also provide protection from infection with other viruses transmitted by *Ae*. *aegypti* (i.e., DENV and ZIKV). These findings complement those regarding the observed effect of the AGO trap in reducing mosquito density and restricting CHIKV infection in mosquitos from the same communities. AGO traps are a novel, chemical-free, effective approach to control *Ae*. *aegypti* populations and provide protection from infection with the pathogens that these mosquitos transmit. Future evaluations should determine if AGO traps are sustainable and effective in larger scale community trials.

## Supporting information

S1 Fig Aerial view of communities with (intervention) or without (non-intervention) autocidal gravid ovitraps in Salinas and Guayama, Puerto Rico. The study sites are enclosed in red.
(DOCX)

S2 Fig Image and schematic of an Autocidal Gravid Ovitrap used to attract and capture female *Aedes aegypti* mosquitos. (DOCX)

S1 Table Census data for communities with (“intervention”) or without (“non-intervention”) autocidal gravid ovitraps in Salinas and Guayama, Puerto Rico, and approach to proportionally enroll 712 study participants using number of enrolled households as the primary sampling unit. (DOCX)

S2 Table Number of autocidal gravid ovitraps traps used for mosquito control and surveillance in intervention and nonintervention communities, Salinas and Guayama, Puerto Rico. (DOCX)

S3 Table Census data of demographic and household characteristics of residents and survey participants of a chikungunya virus seroprevalence survey conducted among communities with (intervention) or without (non-intervention) autocidal gravid ovitraps in Puerto Rico, November 2015–February 2016. (DOCX)

S4 Table Characteristics of households selected to participate in a chikungunya virus seroprevalence survey conducted among communities with (“intervention”) or without (“non-intervention”) autocidal gravid ovitraps in Puerto Rico, November 2015–February 2016. (DOCX)

S1 Checklist STROBE Checklist. (DOC)
