# Supplementary material for: Autocidal gravid ovitraps protect humans from chikungunya virus infection by reducing Aedes aegypti mosquito populations
Source: PLoS Negl Trop Dis. 2019 Jul 25;13(7):e0007538. doi: 10.1371/journal.pntd.0007538 (PMC6657827; doi:10.1371/journal.pntd.0007538)

**Supporting Figure 1** Aerial view of communities with (intervention) or without (non-intervention) autocidal gravid ovitraps in Salinas and Guayama, Puerto Rico. The study sites are enclosed in red.


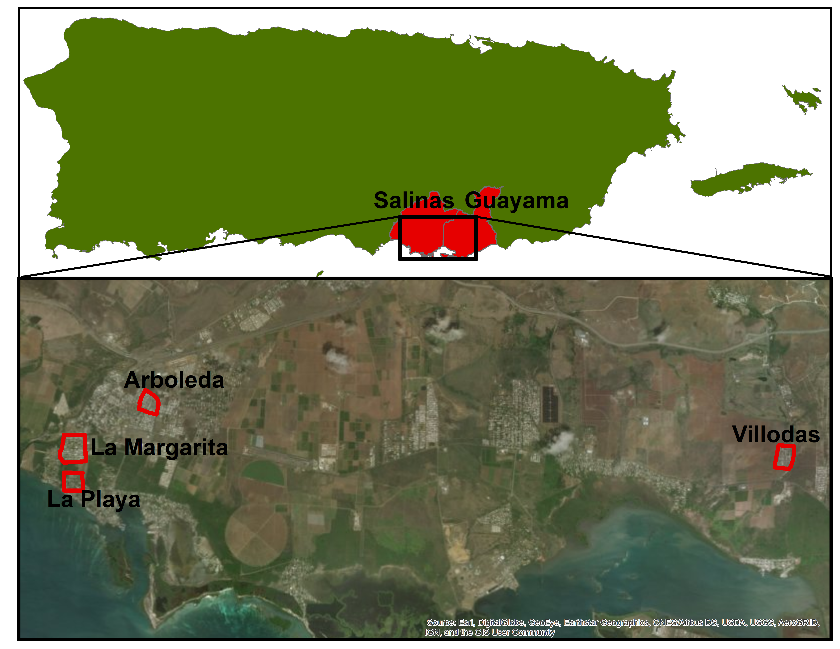

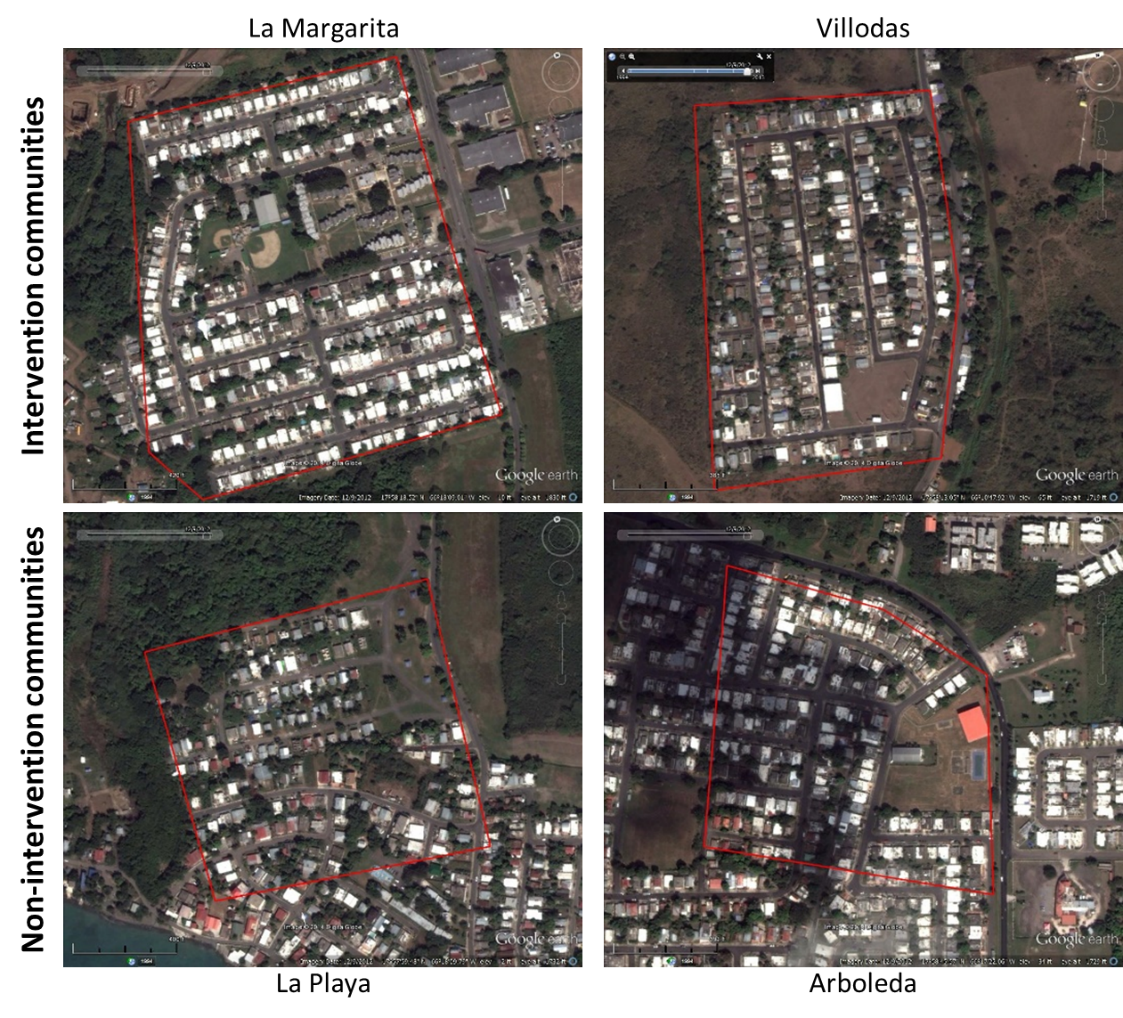

Supplement: S1 Fig — The study sites are enclosed in red. (DOCX) [file pntd.0007538.s001.docx]
