# Supplementary material for: Autocidal gravid ovitraps protect humans from chikungunya virus infection by reducing Aedes aegypti mosquito populations
Source: PLoS Negl Trop Dis. 2019 Jul 25;13(7):e0007538. doi: 10.1371/journal.pntd.0007538 (PMC6657827; doi:10.1371/journal.pntd.0007538)
Supplement: S2 Fig — (DOCX) [file pntd.0007538.s002.docx]

**Supporting Figure 2** Image and schematic of an Autocidal Gravid Ovitrap used to attract and capture female *Aedes aegypti* mosquitos.

**
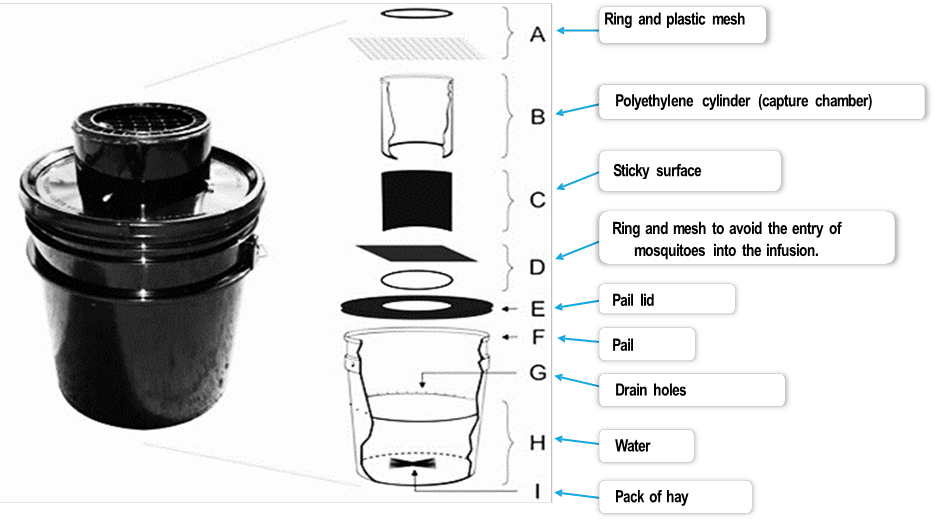
**
