# Supplementary material for: Autocidal gravid ovitraps protect humans from chikungunya virus infection by reducing Aedes aegypti mosquito populations
Source: PLoS Negl Trop Dis. 2019 Jul 25;13(7):e0007538. doi: 10.1371/journal.pntd.0007538 (PMC6657827; doi:10.1371/journal.pntd.0007538)
Supplement: S1 Table — (DOCX) [file pntd.0007538.s003.docx]

**Supporting Table 1** Census data for communities with (“intervention”) or without (“non-intervention”) autocidal gravid ovitraps and approach to proportionally enroll 712 study participants using number of enrolled households as the primary sampling unit, Salinas and Guayama, Puerto Rico.

| **Study group** | **Neighborhood** | **Elevation (meters)** | **Area (hectares)** | **Estimated population (2010)** | **Number of buildings to be visited** | **Target number of households to be offered enrollment** | **Target number of households to be enrolled** | **Average number of residents per household**  **(2010)** | **Target number of individuals to be enrolled** |
| --- | --- | --- | --- | --- | --- | --- | --- | --- | --- |
| Intervention Community | La Margarita | 3 | 18 | 579 | 327 | 126 | 63 | 2.6 | 165 |
|  | Villodas | 20 | 11 | 639 | 241 | 102 | 51 | 3.6 | 182 |
| Nonintervention Community | La Playa | 10 | 17 | 484 | 269 | 106 | 53 | 2.6 | 137 |
|  | Arboleda | 1 | 21 | 800 | 398 | 168 | 84 | 2.7 | 228 |
