# Supplementary material for: Autocidal gravid ovitraps protect humans from chikungunya virus infection by reducing Aedes aegypti mosquito populations
Source: PLoS Negl Trop Dis. 2019 Jul 25;13(7):e0007538. doi: 10.1371/journal.pntd.0007538 (PMC6657827; doi:10.1371/journal.pntd.0007538)
Supplement: S2 Table — (DOCX) [file pntd.0007538.s004.docx]

**Supporting Table 2** Number of of autocidal gravid ovitraps traps used for mosquito control and surveillance in intervention and nonintervention communities, Salinas and Guayama, Puerto Rico.

| **Study group** | **Community** | **# structures** | **# (%) structures with control traps*** | **Estimated # (%) structures occupied and with control traps*** | **# control traps present*** | **# surveillance traps present*** |
| --- | --- | --- | --- | --- | --- | --- |
| Intervention communities | La Margarita | 327 | 281 (88) | 260 (93) | 793 | 40 |
|  | Villodas | 241 | 201 (84) | 168 (82) | 570 | 22 |
| Nonintervention communities | Playa | 269 | N/A | -- | -- | 24 |
|  | Arboleda | 398 | N/A | -- | -- | 29 |

*Numbers varied over time
