# Supplementary material for: Autocidal gravid ovitraps protect humans from chikungunya virus infection by reducing Aedes aegypti mosquito populations
Source: PLoS Negl Trop Dis. 2019 Jul 25;13(7):e0007538. doi: 10.1371/journal.pntd.0007538 (PMC6657827; doi:10.1371/journal.pntd.0007538)
Supplement: S3 Table — (DOCX) [file pntd.0007538.s005.docx]

**Supporting Table 3** Census data of demographic and household characteristics of residents and survey participants of a chikungunya virus seroprevalence survey conducted among communities with (intervention) or without (non-intervention) autocidal gravid ovitraps in Puerto Rico, November 2015–February 2016.

|  | **All households**  **(N = 233)** | **Intervention households**  **(N = 121)** | **Non-intervention households**  **(N = 112)** | ***P* value*** |
| --- | --- | --- | --- | --- |
| **Number residents per household, median (range)** | 2 (1–7) | 2 (1–7) | 2 (1–5) | 0.6895 |
| Number of participants per household, median (range) | 1 (1–5) | 1 (1–4) | 1 (1–5) | 0.2538 |
| Proportion of participants per household, median % (range) | 75 (14–100) | 100 (14–100) | 67 (20–100) | 0.4685 |
| **Total number of residents** | 513 | 273 | 240 | -- |
| Male sex, n (%) | 232 (45.2) | 119 (43.6) | 113 (47.1) | 0.48 |
| Age of residents, median (range) | 53 (5–97) | 52 (5-89) | 56 (5-97) | **<0.0001*** |
| Residents aged <5 years, n (%) | 31 (6.0) | 18 (6.6) | 13 (5.4) | 0.71 |
| Study participants, n (%) | 327 (63.7) | 175 (64.1) | 152 (63.3) | 0.9355 |
| Male participants, n (%) | 122 (52.6) | 63 (52.9) | 59 (52.2) | 1.00 |

*Pearson’s Chi-square was used to compare proportions, and Mann-Whitney-Wilcoxon test was used to test medians

| **Study Group** | **Residents** | **Participants** | ***P* Value*** |
| --- | --- | --- | --- |
| Intervention, median age (range) | 52 (5-89) | 55 (5-89) | **0.0361** |
| Non-Intervention, median age (range) | 56 (5-97) | 58 (6-86) | **0.0309** |

*Wilcoxon Test for difference between medians
