# Supplementary material for: Autocidal gravid ovitraps protect humans from chikungunya virus infection by reducing Aedes aegypti mosquito populations
Source: PLoS Negl Trop Dis. 2019 Jul 25;13(7):e0007538. doi: 10.1371/journal.pntd.0007538 (PMC6657827; doi:10.1371/journal.pntd.0007538)
Supplement: S4 Table — (DOCX) [file pntd.0007538.s006.docx]

**Supporting Table 4** Characteristics of households selected to participate in a chikungunya virus seroprevalence survey conducted among communities with (“intervention”) or without (“non-intervention”) autocidal gravid ovitraps in Puerto Rico, November 2015–February 2016.

| **Characteristics** | **TOTAL**  **(N = 233)** | **Intervention Communities (N=121)** | **Non-Intervention Communities**  **(N=112)** | ***P* value*** |
| --- | --- | --- | --- | --- |
| **Housing type, n (%)^†^** | | | | |
| 1-story house | 216 (92.7) | 113 (93.4) | 103 (92.0) | 0.87 |
| 2-story house | 16 (6.9) | 8 (6.6) | 8 (7.1) | 1.00 |
| Other | 1 (0.4) | 0 (0) | 1 (0.9) | -- |
| **Intact screens on windows and doors, n (%)** | | | | |
| None | 40 (17.2) | 18 (14.9) | 22 (19.6) | 0.43 |
| Some | 77 (33.0) | 47 (38.8) | 30 (26.8) | 0.07 |
| All | 116 (49.8) | 56 (46.3) | 60 (53.6) | 0.33 |
| **Air conditioning use, n (%)** | | | | |
| Never | 87 (37.3) | 50 (41.3) | 37 (33.0) | 0.24 |
| Ever | 146 (62.7) | 71 (58.7) | 75 (67.0) | 0.24 |
| **Leave doors/windows open, n (%)** | | | | |
| Never | 58 (24.9) | 32 (26.4) | 26 (23.2) | 0.68 |
| Ever | 175 (75.1) | 89 (73.6) | 86 (76.8) | 0.68 |
| **Use of mosquito coil, n (%)** | 32 (13.7) | 11 (9.09) | 21 (18.8) | 0.05 |
| **Use of citronella candles, n (%)** | 44 (18.9) | 17 (14.1) | 27 (24.1) | 0.07 |
| **Use of mosquito coil or citronella candles, n (%)** | 60 (25.8) | 22 (18.2) | 38 (33.9) | **<0.01** |
| **Annual income, n (%)** | | | | |
| < $25,000 | 153 (65.7) | 80 (66.1) | 73 (65.2) | 0.99 |
| $26,000–$50,000 | 38 (16.3) | 19 (15.7) | 19 (17.0) | 0.93 |
| > $51,000 | 9 (3.9) | 5 (4.1) | 4 (3.6) | 1.00 |
| Declined to answer | 33 (14.2) | 17 (14.0) | 16 (14.3) | 1.00 |
| *Pearson’s chi-square test for comparison of proportions  ^†^Excludes one non-intervention household of “Other” structure | | | | |
